# Faecal immunochemical test: challenges and opportunities for cancer diagnosis in primary care

**DOI:** 10.3399/bjgp22X720209

**Published:** 2022-07-29

**Authors:** Mary Craig, Jeff Turner, Jared Torkington, Tom Crosby

**Affiliations:** Wales Cancer Network, Cardiff; GP Cancer Lead, Aneurin Bevan University Health Board, Newport.; Wales Cancer Network; Consultant Gastroenterologist, University Hospital Llandough, Cardiff.; University Hospital of Wales, Cardiff.; Velindre University NHS Trust; National Cancer Clinical Director for Wales; Clinical Lead Transforming Cancer Services, Velindre Cancer Centre, Cardiff.

Colorectal cancer (CRC) disproportionately causes the second highest number of UK cancer deaths (16 600 annually) as it has the fourth highest incidence.^1^ The UK has the lowest 1- and 5-year bowel cancer survival rates among the International Cancer Benchmarking Partnership countries,^2^ the majority of patients being diagnosed at late stage (III and IV). The incidence in those aged ≤50 years is increasing (particularly in 20–29-year-olds where incidence increased by 7.9% a year from 2004 to 2016).^3^

Early-stage (I and II) bowel cancer confers 92% 5-year survival,^1^ and optimising faecal immunochemical test (FIT)-based bowel screening is vital to improve bowel cancer outcomes. An observational study found FIT-based screening programmes reduced participant bowel cancer mortality by 41%, dwarfing the 16% reduction demonstrated by guaiac faecal occult blood screening.^4^ Many GP practices are supporting bowel screening by systematically and/or opportunistically giving non-responders encouragement to participate.

Endoscopy capacity has not kept pace with demand. The advent of bowel screening, the National Institute for Health and Care Excellence’s (NICE) lowering of referral thresholds (to attain early-stage diagnosis),^5^ and infection control restrictions/workforce levels due to the pandemic have all contributed to a crisis in endoscopy capacity. This could worsen as bowel screening uptake improves and eligibility is extended to those aged 50 years at a FIT positivity threshold of 80 µHb/g over the next few years.

## FIT ENDORSEMENT AND COVID-19

When NICE endorsed FIT,^6^ it was predicted that FIT could safely hone demand on colonoscopy services, identifying high-risk patients and providing them with earlier diagnosis while reassuring those at very low risk. Rollout of DG30^6^ in the UK has been patchy and evaluation clouded by COVID-19 pathway changes.

COVID-19 resulted in widespread adoption of FIT as a secondary care prioritisation tool, allocating precious endoscopy slots primarily to FIT-positive patients. This resulted in a large-scale natural experiment with very positive results. Modelling of FIT-based triage during COVID-19 concluded that it reduced mortality (attributable to presentation/diagnostic delay) by 89%.^7^

Increasingly, we are seeing FIT being used as a rule-in test/downgrade tool for high-risk patients. In the absence of evidence-based national guidance, this can be concerning for GPs. Furthermore, despite FIT’s usefulness as a triage tool, FIT-based triage can result in delay and inefficient colorectal pathways — decisions are being deferred in the hope of receiving a FIT result; a positive FIT result, received after investigations have started, can lead to additional tests being deemed necessary.

In short, despite FIT’s considerable potential to revolutionise early diagnosis of colorectal cancer, its absence at time of vetting is a source of frustration for triaging consultants, and the exclusion of NG12 criteria patients from urgent investigation because FIT is negative or absent is a source of frustration for GPs.

## WHY SHOULD WE EMBRACE FIT IN PRIMARY CARE?

All bowel symptoms have poor sensitivity and specificity for cancer, making the decision to refer from primary care harder.^8^ In addition, Juul *et al* point out that 50% of CRCs do not present with bowel red flags at all. In their study, 9.4% of their FIT-positive, non-red-flag patients were found to have CRC.^9^

Referral delays of 3 months (for patients with bowel red flags) are associated with significantly worse prognosis than those referred within 2 weeks,^10^ so we need FIT to identify high-risk patients early. Crucially, FIT positivity has greater sensitivity than any single symptom/combination of symptoms for flagging possible CRC.^11^

Saw *et al*’s meta-analysis^12^ evaluated 15 prospective cohort studies (28 832 symptomatic patients) where the FIT result was corroborated by either colonoscopy or 24 months’ follow-up. With the usual FIT threshold of ≥10 µHb/g faeces, FIT has a sensitivity of 88.7% (95% confidence interval [CI] = 85.2 to 91.4) and a specificity of 80.5% (95% CI = 75.3 to 84.8) for CRC. FIT at the lowest limits of detection (≥2 µHb/g) has a sensitivity of 96.8% (95% CI = 91.0 to 98.9) and a specificity of 65.6% (95% CI = 59.0 to 71.6). Juul *et al* also found that 66.7% of primary care FIT-positive CRCs were diagnosed at early stage.^9^ This suggests that universally available symptomatic FIT in primary care could improve patient outcomes.

The highest sensitivity FIT thresholds miss fewer bowel cancers but at a cost of exposing many more to the risks of endoscopy (without finding pathology) and amplifying the gap between endoscopy demand and capacity. It must be remembered though that, at a FIT threshold of ≥10 µHb/g, 11.3% of cancers may be missed.^12^ FIT is an adjunct to — not a replacement for — history, examination, and review of ongoing symptoms.

## NEW GUIDELINES

The Association of Coloproctology of Great Britain and Ireland in conjunction with the British Society of Gastroenterology (ACPGBI/BSG) have just issued national FIT guidance based on consensus statements (including primary care views) backed by extensive evidence review.^13^ The guidance clarifies a number of important clinical issues and is welcomed. It differs from NICE DG30 in that DG30 did not approve FIT in the presence of overt rectal bleeding, but recent evidence finds FIT’s sensitivity for bowel cancer is highest in this context.^12^^,^^14^ The ACPGBI/BSG guideline recommends FIT be used in rectal bleeding as whole-colon imaging (colonoscopy or computed tomography colonography) is needed for FIT-positive rectal bleeders. FIT-negative rectal bleeding is more likely to be ano-rectal as FIT quantifies the degradation products of haemoglobin. Flexible sigmoidoscopy is appropriate for people with FIT-negative significant rectal bleeding.

The guidance also reassures us that, despite early publications raising concern about false-negative FIT CRCs in the presence of iron deficiency anaemia, D’Souza *et al*^11^ provide reassurance that FIT has merit in this patient group too.

FIT is less reliable at alerting (non-cancer) ‘advanced colorectal neoplasia’ (sensitivity 68.4%) and ‘serious bowel disease’, for example, inflammatory bowel disease (sensitivity 69.7%) at ≥10 µHb/g faeces.^12^ Therefore, for patients and their primary care teams seeking to understand and resolve symptoms, access to timely non-2-week wait/urgent suspected cancer specialist advice and/or management with appropriate additional tests must be available for those who test negative.^13^

## CONCLUSION

In relying on FIT to determine the speed, nature, and necessity of investigation, the ACPGBI/BSG guidance strongly advocates that FIT is completed at the earliest opportunity, which is in primary care, preferably prior to referral. We need to ensure symptomatic FIT is fully established in primary care. We need to promote equity of uptake across patient demographic groups. We must understand the barriers to patient concordance and have strategies to address them. Secondary care providers need to retain alternative urgent pathways for high-risk patients who cannot, or do not, complete FIT. Understanding, through effective communication, is essential between patients and health professionals about the shared responsibility for timely investigation when FIT is recommended. Practical safety-netting guidance is needed to support FIT-negative patients in the community. Safety-netting resources are expected from the ACPGBI/BSG group shortly.^13^

NICE intend to update their guidance on symptomatic FIT by December 2023.^15^ In the meantime, we should celebrate and adopt with confidence this pre-test technology that is acceptable to most patients, prudent, and improves early detection of bowel cancer.
